# Supplementary material for: Predicting visual function by interpreting a neuronal wiring diagram
Source: Nature. 2024 Oct 2;634(8032):113–23. doi: 10.1038/s41586-024-07953-5 (PMC11446822; doi:10.1038/s41586-024-07953-5)
Supplement: Supplementary file 6 — CRF and ERF predictions for individual TmY4 and TmY9 cells. Analogous to Supplementary Data 3, but for TmY target types. Shown are the top four monosynaptic pathways, the strongest pathway passing through each of the top ten intermediary types (ranking from Extended Data Fig. 7), and the trisynaptic pathway Tm1–TmY–Dm3–TmY (see the section entitled Prediction of spatial normalization). [file 41586_2024_7953_MOESM6_ESM.zip › DataS4/TmY9qΓèÑ/720575940612183758.pdf]

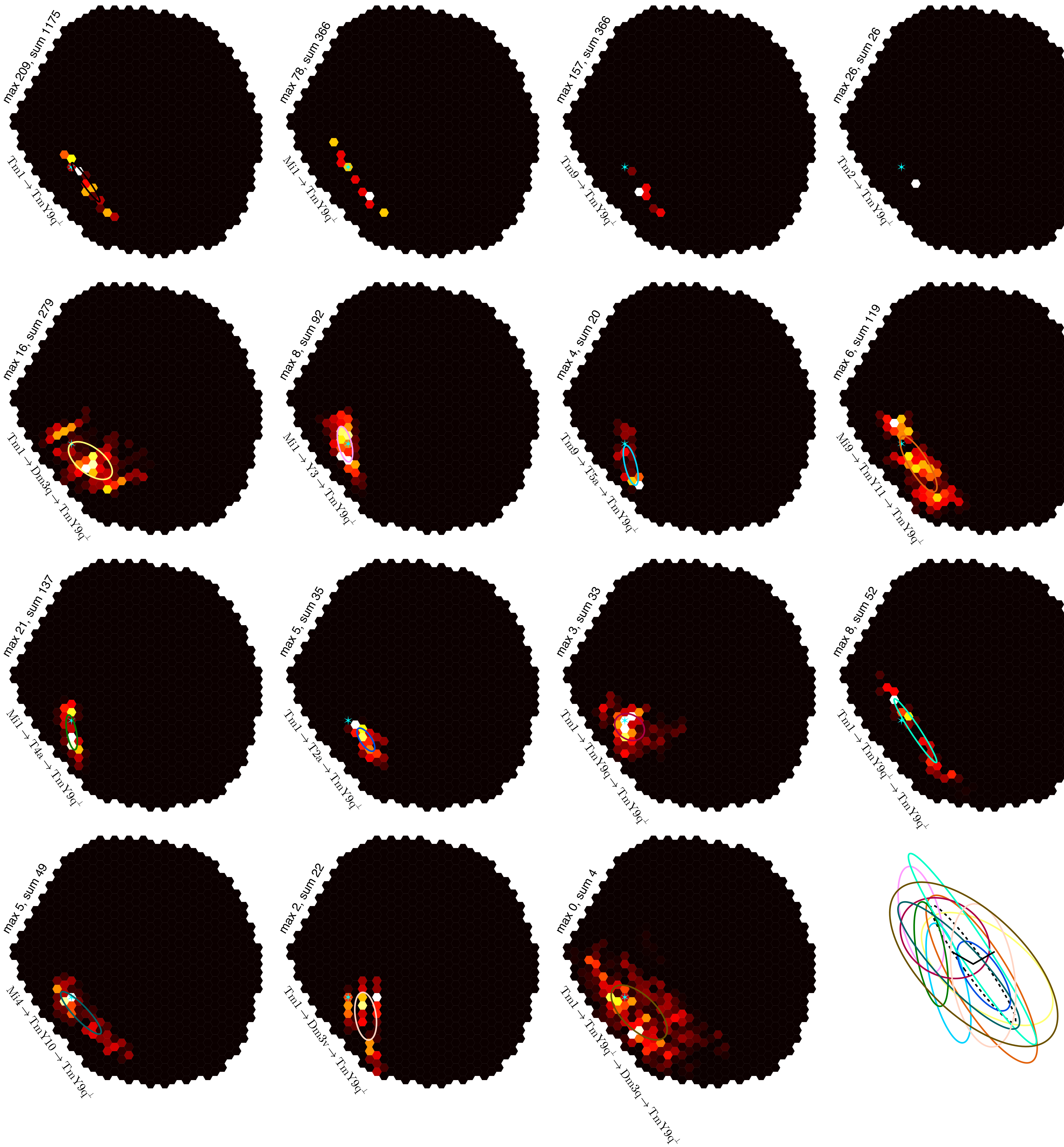
max 209, sum 1175
Tm1 → TmY9q⊥
max 78, sum 366
Mi1 → TmY9q⊥
max 157, sum 366
Tm9 → TmY9q⊥
max 26, sum 26
Tm2 → TmY9q⊥
max 16, sum 279
Tm1 → Dm3q → TmY9q⊥
max 8, sum 92
Mi1 → Y3 → TmY9q⊥
max 4, sum 20
Tm9 → T5a → TmY9q⊥
max 6, sum 119
Mi9 → TmY11 → TmY9q⊥
max 21, sum 137
Mi1 → T4a → TmY9q⊥
max 5, sum 35
Tm1 → T2a → TmY9q⊥
max 3, sum 33
Tm1 → TmY9q → TmY9q⊥
max 8, sum 52
Tm1 → TmY9q⊥ → TmY9q⊥
max 5, sum 49
Mi4 → TmY10 → TmY9q⊥
max 2, sum 22
Tm1 → Dm3v → TmY9q⊥
max 0, sum 4
Tm1 → TmY9q⊥ → Dm3q → TmY9q⊥
